# Uncovering the role of directed connectivity in alpha and theta band activity for sustaining perception-action links

**DOI:** 10.1038/s42003-025-08601-y

**Published:** 2025-08-02

**Authors:** Elena Eggert, Astrid Prochnow, Nasibeh Talebi, Christian Frings, Alexander Münchau, Christian Beste

**Affiliations:** 1 Cognitive Neurophysiology, Department of Child and Adolescent Psychiatry, Faculty of Medicine, TU Dresden, Dresden, Germany; 2 Cognitive Psychology, University of Trier, Trier, Germany; 3 Institute of Systems Motor Science, University of Lübeck, Lübeck, Germany; 4 German Center for Child and Adolescent Health (DZKJ), Partner Site Leipzig/Dresden, Dresden, Germany

**Keywords:** Cognitive control, Neuroscience, Human behaviour

## Abstract

Central to the process of efficient response selection, the integration of perception and action remains a primary focus in neuroscience. The current study sets out to examine the roles of theta, alpha and beta frequency band activity in perception-action binding processes, as well as the corresponding directed connectivity patterns between the associated neuroanatomical structures. To this end, electroencephalography (EEG) data are collected from N = 43 healthy participants performing a classic prime-probe experimental paradigm which are subsequently subjected to EEG-beamforming methods as well as Non-linear Causal Relationship Estimation by Artificial Neural Network in order to identify linear and non-linear connectivity patterns. The results highlight the integral role of the alpha frequency band in the management of perception-action associations, particularly in the maintenance of these associations over time. In contrast, theta band activity appears to be crucial for the organization of sequential information but does not sustain the continuity of perception-action associations across time.

## Abstract (web summary)

Perception-action integration is related to connectivity strength in the alpha frequency band reflecting the maintenance of associations over time, and in the theta frequency band reflecting the organization of sequential information.

## Introduction

Adaptive behavior requires flexible management of perceptual and motor information. In several theoretical approaches, it has been suggested that integrating features pertaining to incoming sensory information and motor responses^1^ plays a key role in these processes^2,3^. The impact of such integrated (bound) feature representations becomes obvious when just recently formed perception-action associations have to be modified during response selection. Several frameworks have described the mechanisms underlying perception-action integration dynamics: The Theory of Event Coding (TEC)^2^ posits that perception and action share a common representational foundation, where sensory and motor features are integrated into event files to facilitate perception-action binding. Building on this, the Binding and Retrieval in Action Control (BRAC) framework^4^ emphasizes the dynamic binding of task-relevant features and their retrieval during action control, highlighting the role of cognitive processes in maintaining and adapting these associations as well as the impact of sensorimotor integration on later processes. Specifically, the history of recently formed perception-action associations is reflected in response selection processes later on and can facilitate or compromise it^4^. Therefore, in prime-probe paradigms capturing these dynamics, a performance decline is observed when stimulus features change from prime to probe while the response that has to be executed remains the same, or vice versa, in comparison to prime-probe combinations in which both the stimulus and the response are repeated^5,6^. The described frameworks provide conceptual models for—and in turn are further informed and substantiated by research findings regarding the neural mechanisms underlying perception-action integration, which has come into focus in recent years^6–13^. One research area involves the conceptualization of oscillatory activity^14–17^, where it has been suggested that especially theta, alpha, and beta band activity plays an important role in the context of perception-action binding and serves distinct functions^18^: Theta band activity likely reflects ongoing integration (binding) and retrieval processes of newly formed perception-action associations, alpha band activity likely modulates/controls these theta band-associated processes, and beta band activity probably serves the storage of perception-action associations. Yet, further research is needed regarding the directed communication between cortical regions associated with theta, alpha, and beta band activity— specifically, when a response in a given situation requires the modification of a previously established perception-action association. This understanding is crucial for identifying feedforward and feedback loops and a possible hierarchical organization underlying adaptive behavior. Working towards this understanding is feasible because of accumulating evidence that the flexible management of perceptual and motor information requires multi-site communication^2,8,18,19^. According to BRAC, the immediate past has an impact on current behavior, which can be guided by response selection processes^4^. In the context of the present study, it can therefore be assumed that (i) cortical structures associated with theta, alpha, and beta frequency bands and (ii) the directed communication between the involved structures during response selection in these frequency bands, should reveal systematic interdependencies with the directed communication pattern evident when an initial association between some perceptual and motor features is established.

To test these assumptions, we employ an established prime-probe paradigm, for which we expect the typical outcome^5,6^, while we apply a data-driven approach on the basis of EEG data. Applying EEG-beamforming methods^20^, we first identify the cortical structures associated with activity in theta, alpha, and beta frequency bands in a task where perception-action bindings are first established (“prime” time point, S1) and later either fully or only partly re-used (“probe” time point, S2). Previous findings suggest that temporo-parietal and insular structures are critical during perception-action integration^8,21,22^. We expect similar brain structures in the aforementioned regions to be associated with activity in the three frequency bands at the S1 or the S2 time point. However, as the computation of connectivity measures between the brain regions requires the calculation of source estimates within each condition instead of contrasts as done in the previous studies^8,21,22^, it is also possible that other brain regions that, for instance, might be more related to visual processing of the stimuli, are of importance^23^. The directed connectivity pattern between the aforementioned cortical regions examined at the S1 and the S2 time points is revealed using non-linear causal relationship estimation by artificial neural networks (nCREANN)^24–26^. Directed functional connectivity between brain regions is linear and non-linear^27^. Moreover, the integration of perception and action depends on feedforward and feedback loops^28^: Feedback processes refer to online error corrections that generate control signals based on the difference between an action goal and the achieved state, whereas feedforward processes describe internal forward models that are used to predict the state that will be achieved by an action^29,30^. Both processes interact to accomplish successful sensorimotor integration and are based on both linear and non-linear dynamics^31–33^. nCREANN allows to capture linear and non-linear connectivities, which is why this method is used in the current study.

## Results

**Fig. 1:**
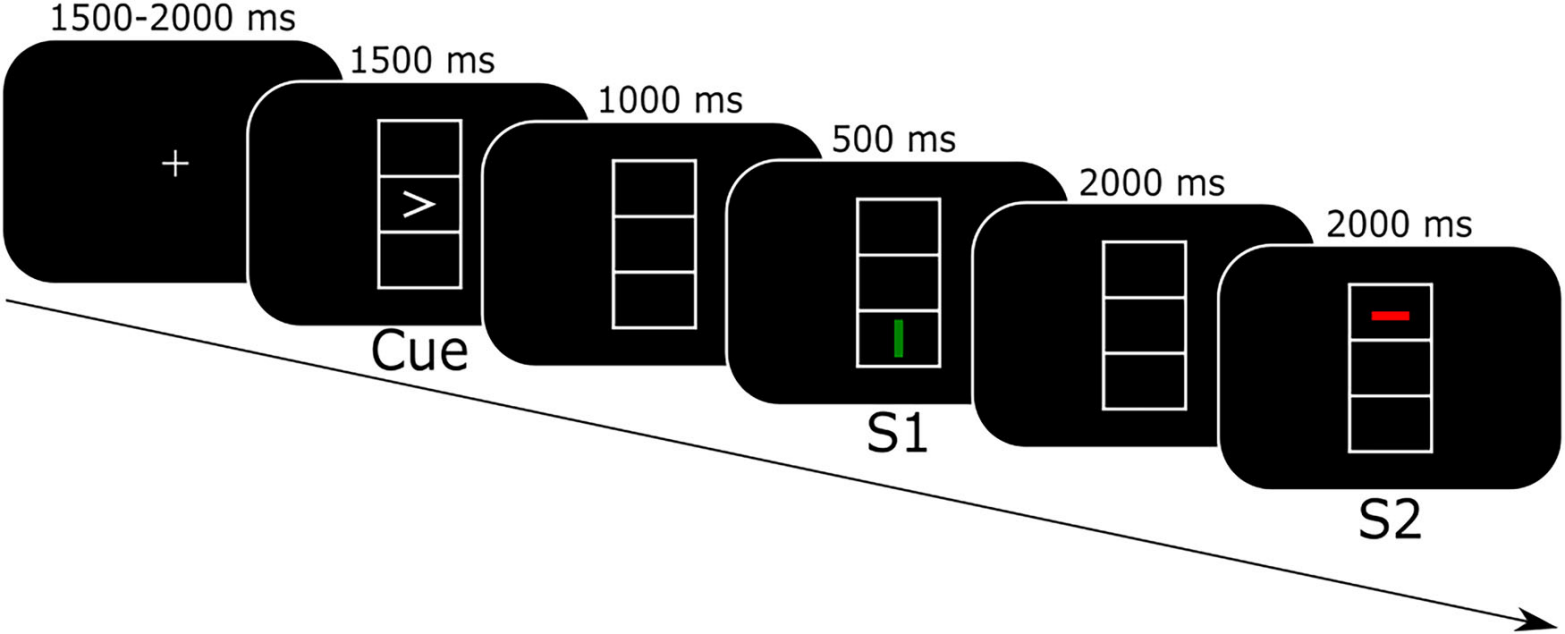
Task illustration. Schematic illustration showing the timing and the stimuli sequence and of the experimental paradigm. The cue is represented by an arrow pointing either left or right, while the consecutively shown stimuli S1 and S2 both constitute a bar that can differ by color (red or green), position (top or bottom), and orientation (vertical or horizontal). The first response is given following S1 and is based on the direction of the cue, while the second response is given following S2 and is based on the orientation of S2.

The used task employed a prime-probe structure as described above, where the prime is referred to as S1 and the probe as S2 in the following. Moreover, the S1 was preceded by a cue. Responses were given after S1, dependent on the direction of the cue, and after S2, dependent on the orientation of S2. S1 and S2 could either share all stimulus features with each other (full overlap) or no stimulus features at all (no overlap). The responses to the two stimuli could either require responding with the same key (response repetition) or with different keys (response alternation). An example trial of the task is displayed in Fig. 1; more details can be found in “Task”.

The behavioral data was analysed statistically by means of repeated-measures ANOVAs in a within-subject design with the hit rate (i.e., number of correct responses divided by total number of events, including misses and false responses) and the reaction time (i.e., the time passed between stimulus presentation and response, where the mean for each condition is calculated per participant) as the measures of interest. The post-hoc analyses, as well as any other comparisons, were conducted using two-tailed paired-samples t-tests. In the event of a lack of normal distribution as determined by a Kolmogorov–Smirnov test, a non-parametric test was used (Wilcoxon signed-rank test).

### Degree of overlap and response type interact in the expected way

A series of repeated-measures ANOVAs with the factors “response” (repetition vs alternation) and “overlap” (no overlap vs full overlap) was carried out. For accuracy, the results showed a significant interaction between the factors “response” and “overlap” (*F*(1,42) = 97.41, *p* < 0.001, *ƞ*_p_² = 0.699), with a higher accuracy rate in the full overlap trials (93.91% ± 6.93) than in the no overlap condition (78.51% ± 13.80) in the repetition trials (*Z* = −5.33, *p* < 0.001). In the alternation trials (*Z* = −5.50, *p* < 0.001), on the other hand, there was a higher accuracy rate in the no overlap condition (95.53% ± 4.50) than in the full overlap condition (78.12% ± 13.67). The main effects of “response” and “overlap” were not significant (all *F* < .96, all *p* > 0.333).

**Fig. 2:**
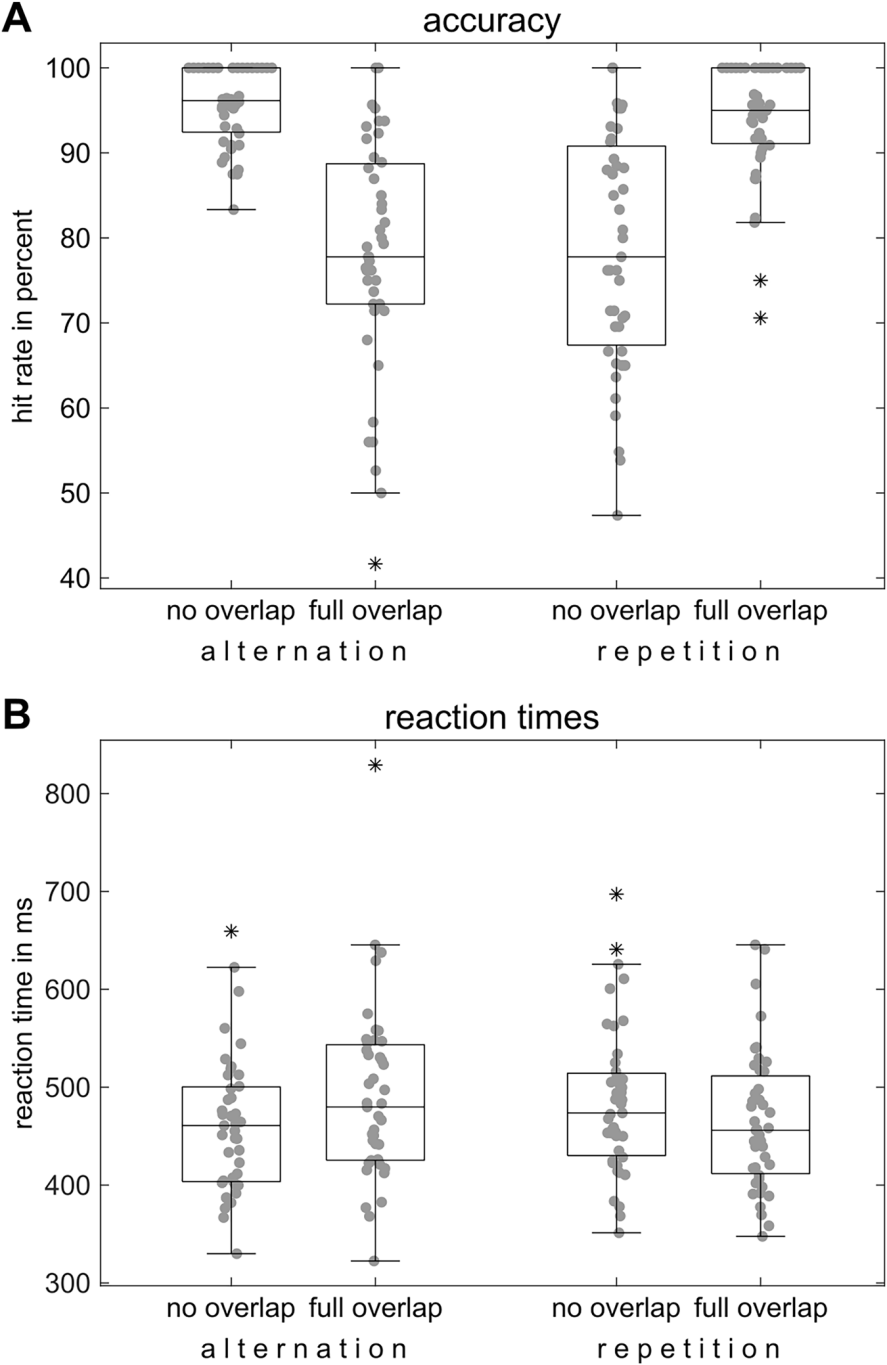
Behavioral results. Boxplots showing **A** hit rates in percent and **B** reaction times in ms with respect to S2 of *N* = 43 participants. The box indicates the interquartile range (IQR) from the first quartile (Q1) to the third quartile (Q3), with the median marked as a horizontal line inside the box. Whiskers extend to the smallest and largest data points within 1.5 × IQR from Q1 and Q3. Individual data points are displayed as gray dots. Outliers are denoted by asterisks.

For the reaction times, the analysis yielded a significant interaction between the factors “response” and “overlap” (*F*(1,42) = 38.670, *p* < 0.001, *ƞ*_p_² = 0.479). Here, the participants responded faster in the full overlap condition (465 ms ± 71) compared to the no overlap condition (485 ms ± 75) in the repetition trials (*t*(42) = 3.65, *p* < 0.001), while the response times were longer in the full overlap condition (491 ms ± 90) than in the no overlap condition (462 ms ± 70) in the alternation trials (*t*(42) = −4.95, *p* < 0.001). The main effects of “response” and “overlap” did not reach the significance level (all *F* < 1.25, all *p* > 0.270). The results of the analysis of the behavioral data are shown in Fig. 2.

To summarize, the behavioral data replicate numerous previous findings. Importantly, the evident interaction of the factors “response” and “overlap” shown for performance accuracy indicates binding effects^2,11,34^.

### Binding and retrieval are reflected in insular and temporal pole theta band activity, insular and lingual gyrus alpha band activity, and lingual gyrus beta band activity

Trials were only included in the analysis of the neurophysiological data if (1) they were correct and (2) there were valid data throughout the whole trial length (see “EEG recording and pre-processing”). On average, 21.9 ± 4.2 alternation trials without overlap, 17.8 ± 4.1 alternation trials with full overlap, 18.0 ± 4.2 repetition trials without overlap, and 21.4 ± 4.3 repetition trials with full overlap were included in the analysis of the neurophysiological data, resulting in 79.1 ± 9.9 trials for the analysis of the post-S1 time window. The neurophysiological analyses were carried out for the theta (4–7 Hz), alpha (8–12 Hz), and beta (15–30 Hz) frequency bands.

**Fig. 3:**
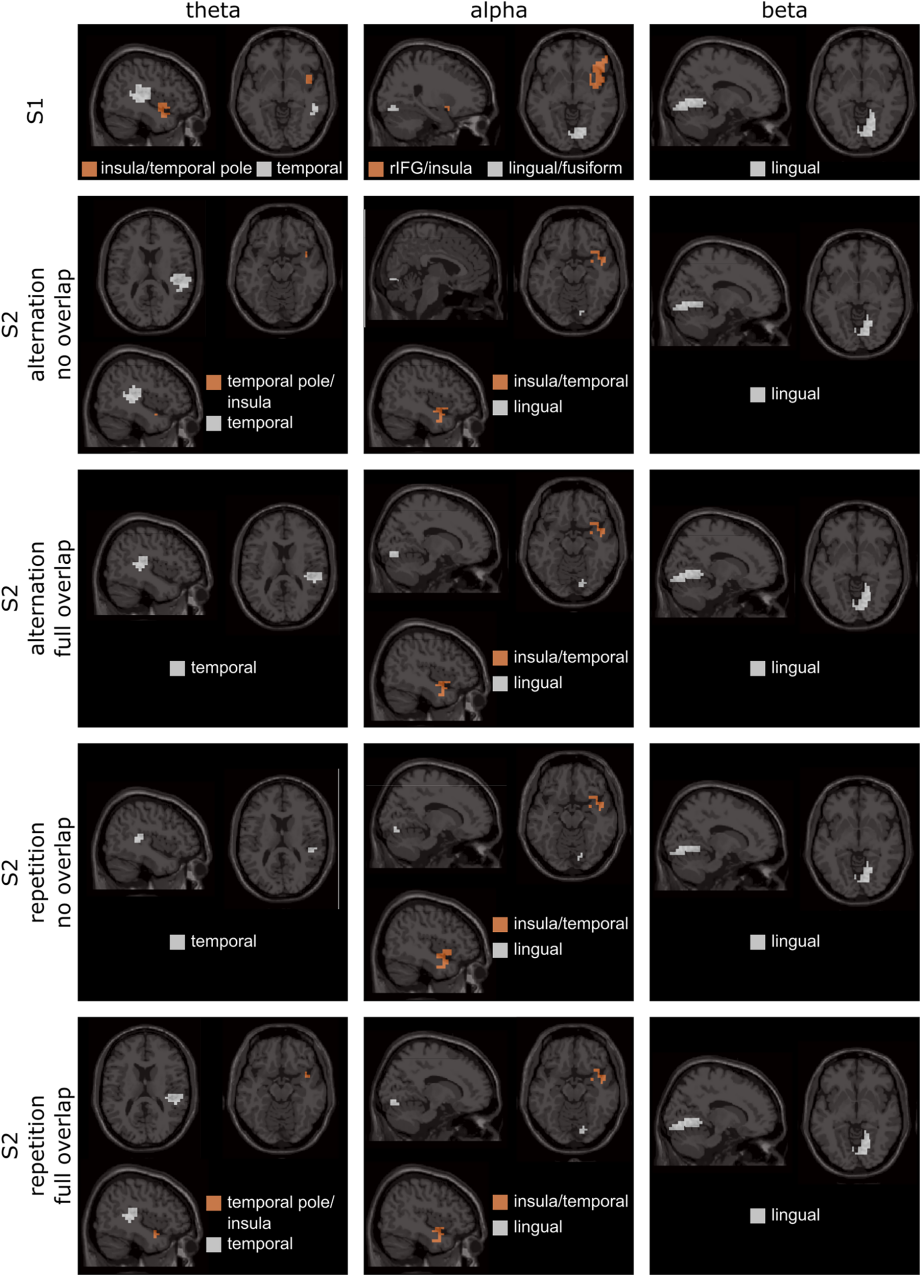
Beamforming results of single conditions. Results of DICS beamforming using the NAI in S1 and each S2 condition (top to bottom) across three frequency bands (left to right). Displayed brain regions represent the top 1% of activity. Calculations were based on *N* = 43 participants.

To estimate directed communication between involved brain regions in the different conditions, single-trial data were used^24–26^. Therefore, the DICS beamforming^20^ and subsequently applied DBSCAN algorithm^35^ were also applied to the single conditions and activity sources in the theta, alpha, and beta frequency bands for the post-S1 time window, as well as each of the conditions in the post-S2 time window based on the Neural Activity Index (NAI) were revealed. An overview of the beamforming results for all three frequency bands can be found in Fig. 3.

In the theta frequency band, the results showed clusters of activity in the insula/temporal pole (BA 13, BA 38) and temporal regions (BA 21, BA 22, BA 39) in the post-S1 time window. In the post-S2 time window in the theta band, there were clusters of activity in the temporal pole/insula (BA 38, BA 13) and the temporal regions (BA 21, BA 22) in the no overlap alternation condition, in the temporal regions (BA 21, BA 22, BA 39) in the full overlap alternation condition, in the temporal regions (BA 21, BA 22, BA 39) in the no overlap repetition condition as well as in the temporal pole/insula (BA 38, BA 13) and temporal regions (BA 21, BA 22) in the full overlap repetition condition. In the alpha frequency band, the results revealed activity clusters in the right inferior frontal gyrus (rIFG)/insula (BA 11, BA 13) and the lingual/fusiform gyri (BA 19, BA 37) in the post-S1 time window. In the post-S2 time window in the alpha band, the results showed activity clusters in the insula/temporal regions (BA 13, BA 21, BA 22, BA 38) and in the lingual gyrus (BA 19) in all four conditions. In the beta frequency band, an activity cluster was revealed in the lingual gyrus (BA 19, BA 38) in the post-S1 time window as well as in all four conditions in the post-S2 time window.

To summarize, post-S1, activity clusters were observed in the insula/temporal pole and temporal regions in the theta band. Post-S2, theta activity varied by condition, showing clusters in the temporal pole/insula and temporal regions. In the alpha band, post-S1 activity appeared in the right inferior frontal gyrus (rIFG)/insula and lingual/fusiform gyri, while post-S2 activity was observed in the insula/temporal and lingual regions across all conditions. For the beta band, clusters emerged in the lingual gyrus post-S1 and post-S2. For a calculation of binding effects on the neurophysiological level (i.e., contrasts between different conditions), please refer to Supplementary Analysis 1 and Supplementary Fig. 1.

### Directed connectivity analysis in the theta and alpha frequency bands

**Fig. 4:**
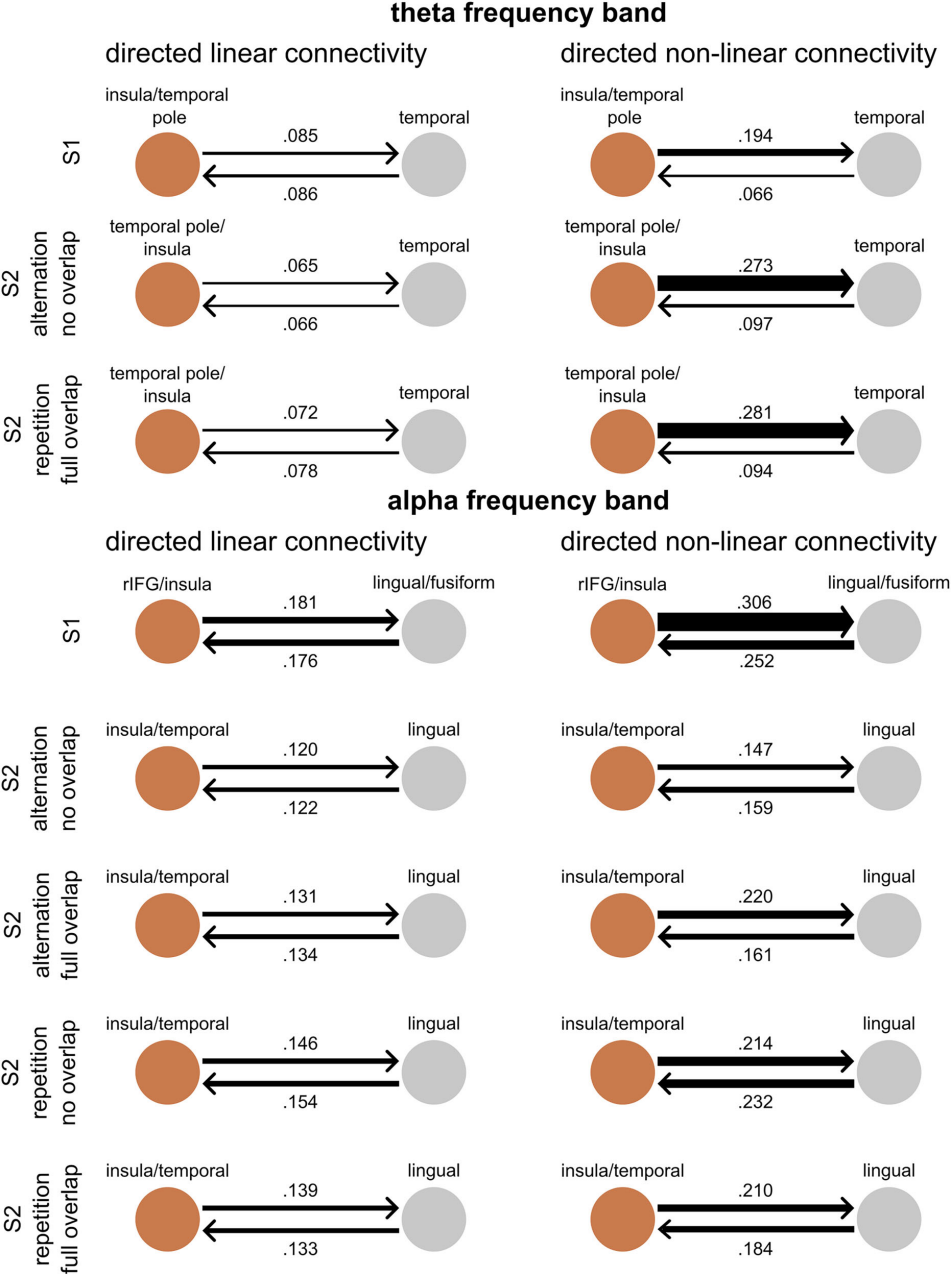
Connectivities based on nCREANN. Connectivity strengths between clusters in the theta and the alpha frequency bands for linear directed connectivity and non-linear directed connectivity. The thickness of the arrows denotes the respective strength of the connectivity (i.e., a thicker arrow represents a higher connectivity value). Calculations were based on *N* = 43 participants.

In the following, the directed connectivity values averaged across subjects in the alpha and theta frequency bands are presented separately for linear and non-linear directed connectivities. A schematic overview of the connectivities, along with the specific connectivity values, is shown in Fig. 4. Note that the connectivity value is a unitless measure.

The neural network demonstrated strong predictive accuracy for all participants. It achieved an R² ≥.941 and an MSE ≤.078 on the training data, and an R² ≥ 0.943 and an MSE ≤ 0.100 on the test data, indicating consistent generalization performance.

Seeing as for the theta band, there was only one activity cluster in two of the post-S2 time window conditions (full overlap alternation, no overlap repetition), connectivity analysis could not be conducted for these conditions, and the following analysis only includes the conditions with two activity clusters for the theta frequency band. In the beta frequency band, there was only one activity cluster in the post-S1 time window and post-S2 conditions, precluding the beta band from any connectivity analysis. Spearman correlations were conducted to examine the relationships between the connectivities in the post-S1 time window and post-S2 conditions for the alpha and the theta band, respectively. Given the large number of correlations, the False Discovery Rate (FDR) correction was applied to control for Type I errors. We employed the Benjamini–Hochberg procedure at a significance level 0.05^36^, establishing critical values for the respective analyses.

### Linear alpha band connectivity strengths are strongly correlated, while linear theta band connectivity strengths are not

Spearman correlations were calculated between all connectivities of the post-S1 time window and post-S2 conditions (i.e., no overlap alternation, full overlap repetition) in the theta band. After applying the FDR correction, resulting in a critical value of 0.007, there was significant correlation between the connectivities within the full overlap repetition condition (*r*_s_ = 0.555, *p* < 0.001). There were no other statistically significant relationships between the connectivities of any of the other conditions in the theta band.

In the alpha band, Spearman correlations were calculated between all connectivities of the post-S1 time window and post-S2 conditions (i.e., no overlap alternation, full overlap alternation, no overlap repetition, full overlap repetition). The FDR correction resulted in a calculated critical value of 0.043. Consequently, all *p*-values below this threshold were deemed statistically significant.

As shown in Table 1, out of 45 correlations tested, 38 remained significant after applying FDR correction. The findings outlined in Table 1 reflect that strong correlations were observed between the post-S1 time window connectivities and all post-S2 conditions. For example, connectivity from the lingual/fusiform gyrus to the rIFG/insula cortex in the post-S1 period correlated significantly with similar connectivities in subsequent trial conditions. Consistent correlations were also noted across the trial types and conditions, indicating stable connectivity patterns in the alpha band between these brain regions.

**Table 1:** Correlations between all conditions for linear connectivities in the alpha frequency band

Variable			*M*	*SD*	1	2	3	4	5	6	7	8	9	10
1	Post-S1	Lingual/fusiform to rIFG/insula	0.176	0.086	-	0.860**	0.514**	0.322*	0.459**	0.569**	0.378*	0.429**	0.424**	0.530**
2	Post-S1	rIFG/insula to lingual/fusiform	0.181	0.080		-	0.578**	0.386*	0.434**	0.574**	0.339*	0.405**	0.403**	0.527**
3	Alternation No overlap	Lingual to insula/temporal	0.122	0.052			-	0.523**	0.390*	0.541**	0.472**	0.334*	0.492**	0.503**
4	Alternation No overlap	Insula/temporal to lingual	0.120	0.058				-		0.350*		0.415**		
5	Alternation Full overlap	Lingual to insula/temporal	0.134	0.048					-	0.451**	0.384*		0.591**	0.484**
6	Alternation Full overlap	Insula/temporal to lingual	0.131	0.056						-	0.524**	0.372*	0.455**	0.482**
7	Repetition No overlap	Lingual to insula/temporal	0.154	0.068							-	0.549**		0.369*
8	Repetition No overlap	Insula/temporal to lingual	0.146	0.067								-		0.449**
9	Repetition Full overlap	Lingual to insula/temporal	0.133	0.063									-	0.563**
10	Repetition full overlap	Insula/temporal to lingual	0.139	0.057										-

^*^ *p* < FDR-threshold of.043, **p < 0.01

To summarize, in the alpha frequency band, significant connectivity was observed between the rIFG/insula and lingual/fusiform gyri in the *post-S1* and for similar activity clusters in the *post-S2* time window, with stable correlations between these time periods. In the theta frequency band, directed connectivity analysis showed varying connectivity strengths between the insula/temporal pole and the temporal regions across different conditions, but no systematic correlations between the analyzed time windows.

### Non-linear connectivity strengths are correlated in the alpha frequency band, but not in the theta frequency band

Spearman correlations were calculated between all connectivities of the post-S1 time window and the post-S2 conditions in the theta band. After applying the FDR correction, no *p* values met the FDR-corrected significance threshold, indicating no statistically significant relationships between the conditions.

Spearman correlations were calculated between all connectivities of the post-S1 time window and the post-S2 conditions in the alpha band, revealing five significant correlations between connectivities, which are shown in Table 2. All other correlations did not reach a significance level after FDR correction.

**Table 2:** Correlations between all conditions for non-linear connectivities in the alpha frequency band

Variable			*M*	*SD*	1	2	3	4	5	6	7	8	9	10
1	Post-S1	Lingual/fusiform to rIFG/insula	0.252	0.197	-	0.672**								
2	Post-S1	rIFG/insula to lingual/fusiform	0.306	0.223		-								.
3	Alternation No overlap	Lingual to insula/temporal	0.159	0.119			-	0.569**						
4	Alternation No overlap	Insula/temporal to lingual	0.147	0.094				-				.		
5	Alternation Full overlap	Lingual to insula/temporal	0.160	0.096					-					
6	Alternation Full overlap	Insula/temporal to lingual	0.220	0.132						-				
7	Repetition No overlap	Lingual to insula/temporal	0.232	0.155							-	0.458**		
8	Repetition No overlap	Insula/temporal to lingual	0.214	0.153								-	0.465**	
9	Repetition Full overlap	Lingual to insula/temporal	0.184	0.147									-	0.482**
10	Repetition Full overlap	Insula/temporal to lingual	0.210	0.137										-

FDR-threshold of.017, **p < 0.01

To summarize, in the alpha frequency band, the analysis revealed five significant correlations within the post-S1 time window and in several post-S2 conditions. In the theta frequency band theta frequency band, no significant correlations were found between the post-S1 and post-S2 time windows.

### Directional asymmetry observed only in non-linear connectivity of alpha and theta bands

**Fig. 5:**
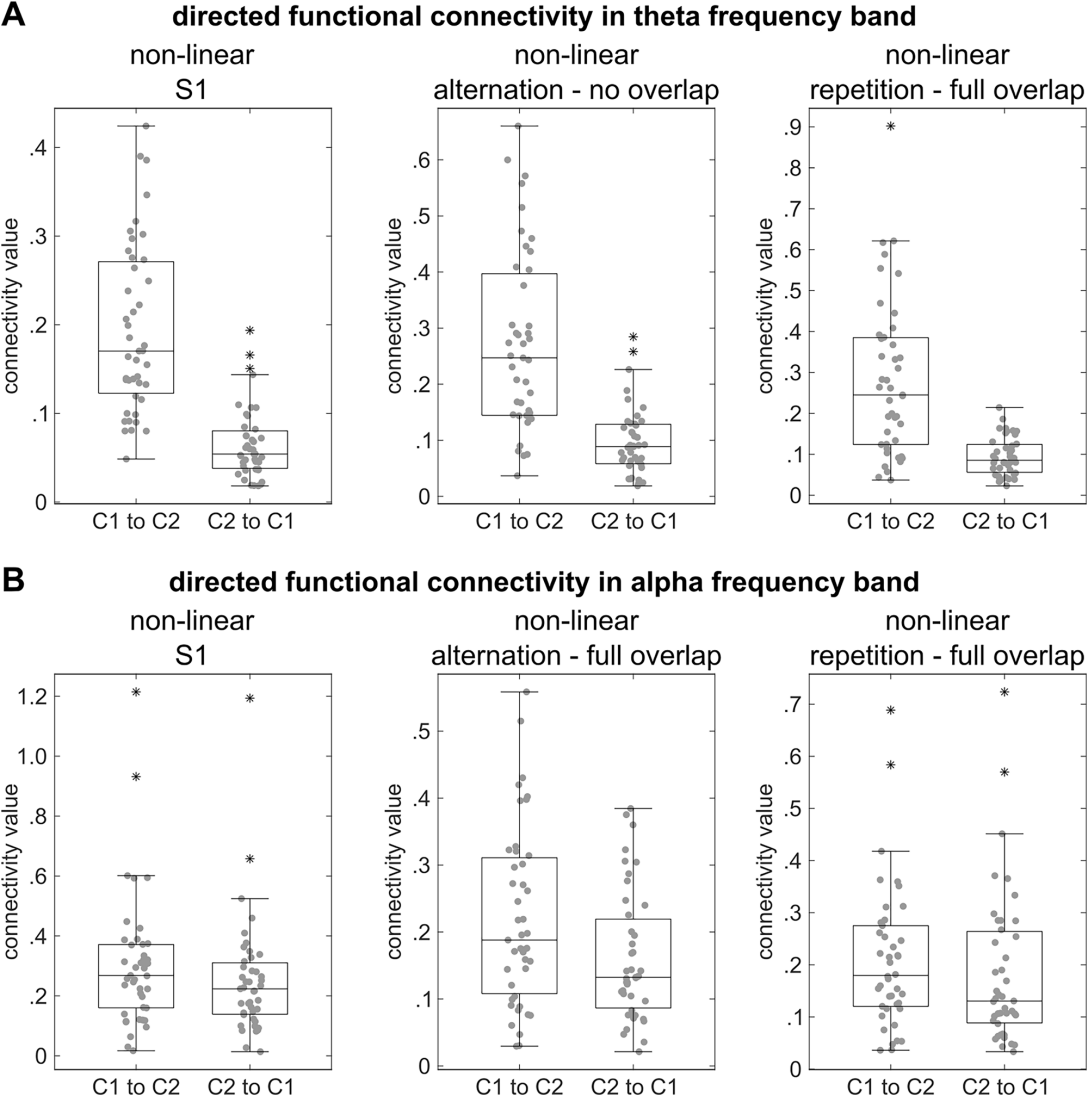
Distribution of nCREANN values for significant comparisons. Figure part **A** shows the distribution of directed functional connectivity values in the theta frequency band of *N* = 43 participants. After S1, cluster 1 (C1) refers to the insula/temporal pole and cluster 2 (C2) to the temporal cortex. After S2, C1 refers to the temporal pole/insula and C2 to the temporal regions. Figure part (**B**) displays the distribution of directed functional connectivity values in the alpha frequency band of *N* = 43 participants. After S1, C1 refers to the right inferior frontal gyrus/insula and C2 to the lingual/fusiform gyrus. After S2, C1 refers to the insula/temporal cortex and C2 to the lingual gyrus. The box indicates the interquartile range (IQR) from the first quartile (Q1) to the third quartile (Q3), with the median marked as a horizontal line inside the box. Whiskers extend to the smallest and largest data points within 1.5 × IQR from Q1 and Q3. Individual data points are displayed as gray dots. Outliers are denoted by asterisks.

An overview of the statistically significant results can be seen in Fig. 5.

For the theta band and the linear directed connectivities, paired-samples *t*-tests were conducted comparing the connectivities between the activity clusters within each condition. None of the comparisons showed a significant difference (all *p* > 0.071). For the non-linear directed connectivities, *t*-tests/Wilcoxon signed-rank tests were conducted to compare the connectivities between the activity clusters within each condition. The connectivity values from the temporal pole/insula to the temporal regions were higher than the connectivity values from the temporal regions to the temporal pole/insula (post-S1 time window: 0.194 ± 0.095 vs. 0.066 ± 0.040, *t*(42) = −9.640, *p* < 0.001; no overlap alternation condition: 0.273 ± 0.159 vs. 0.097 ± 0.061, *Z* = −5.398, *p* < 0.001; full overlap repetition condition: 0.281 ± 0.191 vs. 0.094 ± 0.046, *t*(42) = −6.503, *p* < 0.001).

For the alpha band, and for the linear directed connectivities, none of the comparisons of the connectivities between the activity clusters within each condition showed a significant difference (all *p* > 0.200). Regarding non-linear connectivities in the alpha band, it was shown that there were higher connectivity values from the rIFG/insula to the lingual/fusiform gyri than from the lingual/fusiform gyri to the rIFG/insula in the post-S1 time window (0.306 ± 0.223 vs. 0.252 ± 0.197; *Z* = −1.99, *p* = 0.046), the full overlap alternation condition (0.220 ± 0.132 vs. 0.161 ± 0.096; *Z* = −2.52, *p* = 0.010) and the full overlap repetition condition (0.210 ± 0.137 vs. 0.183 ± 0.147; *Z* = −2.02, *p* = 0.043). None of the other comparisons showed a significant difference (all *p* > 0.469).

The analysis of the variance of the directed connectivity strength across conditions representing the binding effect in the alpha band can be found in the Supplementary Analysis 2. For the theta band, binding effects for the connectivities could not be estimated due to only two of the conditions including more than one activity cluster. To calculate binding effects, however, all four conditions are a prerequisite, which is why the binding effects could only be computed for the alpha frequency band.

## Discussion

The current study examined directed communication in cortical theta, alpha, and beta band activity during the dynamic management of perception (*S*) and action (*R*) associations (bindings). Specifically, we examined to which directed network connectivity patterns, when establishing a perception-action association, relate to network connectivity patterns evident when the same perception-action association is retrieved (and, if required, reconfigured) during response selection. The study was motivated by ideomotor concepts^37,38^ and by recent developments showing that activity in the theta, alpha, and beta frequency bands is involved in distinct processes^18^ related to the binding and retrieval/reconfiguration of integrated perception-action representations^4^.

The behavioral data (Degree of overlap and response type interact in the expected way) provided a replication of well-established effects^4,34^ documenting a binding/association between perceptual and motor processes during response selection^2^: Whenever there was a strong overlap between features of the prime and the probe stimulus, response accuracy decreased and reaction times increased when the response had to be changed. This reflects partial repetition costs, which occur when the previously established stimulus-response bindings and expectancies regarding stimulus-response associations are only partially fulfilled^2^. In contrast, a strong overlap between the S1 and S2 stimulus features improved response accuracy and led to faster reaction times when responses were repeated. These results show that the immediate history affects subsequent response selection processes and are in line with principles of the Theory of Event Coding (TEC)^2^ and binding and retrieval in action control (BRAC)^3,4^.

### Alpha band activity and connectivity reflect top-down control during perception-action integration

Most important are the findings from the directed connectivity analysis based on the results of the neural activity indices (Binding and retrieval are reflected in insular and temporal pole theta band activity, insular and lingual gyrus alpha band activity, and lingual gyrus beta band activity): Analyzing the neural activity indices in the alpha and beta frequency bands revealed consistent cortical activity patterns (Binding and retrieval are reflected in insular and temporal pole theta band activity, insular and lingual gyrus alpha band activity, and lingual gyrus beta band activity). Similar neural activity patterns appeared after S1 (establishment of the perception-action association) and after S2 (retrieval/reconfiguration of the perception-action association), regardless of whether reconfiguration relative to S1 was required. This pattern reflects ideomotor principles^38^ and the principle of binding and retrieval^4^ on a neural level. According to the concept of BRAC^4^, responses to S1 should affect responses to S2, and thus there should be an overlap between the processes involved in binding and those involved in retrieval. This is reflected in the correspondence of neural activity patterns in functional neuroanatomical structures associated with alpha band and beta band activity in ventral stream cortical regions (i.e., lingual/fusiform gyri). The importance of these ventral stream regions is plausible because the dynamics of integrated perception-action associations are strongly determined by the identity of features constituting a stimulus (e.g., color, orientation, size, etc.) that is bound to a specific response^2,39^, and this identity information is processed in the ventral visual processing stream^40^. The fusiform gyrus in particular has been shown to have a role in the recognition and processing of object information related to form^41,42^. Moreover, the lingual gyrus is thought to be involved in both the manipulation of visual information^43^, which might be of relevance during the creation on an event file, as well as in the retrieval of visual and episodic memory^44–46^ such as event files. In addition, there were clusters of activity in the alpha frequency band in the rIFG and insula post-S1, likely reflecting the inhibitory gating of the integration of sensory information into an event file^14,47,48^, and insular and temporal regions post-S2, likely reflecting the retrieval of event file information based on the integrated sensory information from S2^47^. Further substantiating the importance of alpha band activity for ideomotor principles and the understanding of the dynamic management of perception-action associations, there were positive correlations of the linear directed information transfer between the alpha band-associated functional neuroanatomical structures active in the post-S1 and post-S2 time intervals (Directed connectivity analysis in the theta and alpha frequency bands, Table 1). Here, the strength of the directed information transfer between the rIFG/insula and lingual/fusiform gyrus (post-S1) and between the lingual gyrus and the insula/temporal areas (post-S2 conditions) was positively correlated. Thus, the findings indicate that processes during binding (post-S1) affect the processes during retrieval (post-S2) in the alpha band, which is in line with the BRAC principle emphasizing the impact of the immediate past on adaptive behavior^4^. It has been suggested that alpha band activity enables top-down control during the dynamic management of perception-action associations^18^. This is reflected by the directed information transfer shown for alpha band activity. All this evidence suggests that directed communication between brain regions associated with alpha band activity is essential to understand the dynamic management of perception-action associations.

### Theta band activity organizes sequential information but does not drive adaptive behavior influenced by the immediate past

The current data show that alpha band activity, in particular, reflects the impact of the immediate past on adaptive behavior. This is substantiated by the findings from theta band activity, which dissociate from the findings in alpha band activity: After the S1 and thus during the binding process, the most involved regions were the insula, suggested to be an “integration hub” for different sensory information^47^, the temporal pole which is associated with complex visual processing and memory processes^49^, and the gyrus angularis which is considered as an associative area connecting sensory and motor information^50^. This likely reflects the binding of sensory information into an event file, combining sensory input with the respective motor response. During the retrieval (post-S2), however, the activity in the theta frequency band was more focal in the gyrus angularis in the conditions requiring a reconfiguration (alternation full overlap, repetition no overlap), implying an increased need for associating sensory and motor information^50^, while the activity additionally included insula and temporal pole, involved in integrating sensory information^47^ and memory processes^49^, in less difficult conditions (alternation no overlap, repetition full overlap). Although the data show evidence for directed information exchange within the theta band between cortical regions during the integration of retrieval of perception-action associations (Directed connectivity analysis in the theta and alpha frequency bands), there was no general correlational pattern between the activity clusters in the post-S1 and the post-S2 time window. This suggests that theta band activity and directed communication therein are important during integration (binding) and retrieval processes, which aligns with several lines of evidence from perception-action integration^18^ and conceptions that theta activity can integrate processes across distinct cortical regions^51,52^. Yet, theta band-related directed information transfer between cortical regions is unlikely a mechanistic element underlying how the immediate past impacts adaptive behavior and the dynamic management of perception-action associations. Theta band activity underlies the organization of sequentially ordered information, whereas alpha band activity reflects the active inhibition of task-irrelevant information^14,53^. For the immediate past to affect adaptive behavior, it is important that information is maintained and distinguished from other information. The latter seems relevant for and depends on directed information transfer in alpha band activity. Of note, the activity in neither of the frequency bands originates mainly from parietal areas, although they have been frequently found in previous studies^8,21,22^. This likely results from the fact that the current study assessed the NAI instead of contrasting different conditions, which has been the approach in most of the previous studies^8,21,22^. Importantly, also for other paradigms for which previously fronto-parietal areas have been obtained when contrasting conditions^54,55^, a pattern of visual areas has been obtained when assessing the NAI^23^.

### Summary and conclusion

The quintessence of this research has substantial implications for research in action control. The BRAC framework^3,4^ stresses the importance of the immediate past in action control and has revealed that nominally different experimental procedures and tasks tap into the same cognitive mechanisms. According to BRAC, many of the dynamics relevant to understanding action control refer to the impact of the immediate past. Electrophysiological research in the field is dominated by conceptions regarding theta band activity^15,18,56^. The current findings, however, show that alpha band activity is most relevant. The current results call for a shift in research strategy in which directed communication between alpha band-associated cortical regions must be examined further to better understand adaptive behavior. While data-driven approaches can be highly effective, they may also be considered a limitation due to their reliance on the available dataset, which can restrict both generalizability and interpretability. To address this concern in our neural network analysis, we divided the dataset into separate training and testing subsets to explicitly evaluate the model’s generalization performance. Although the number of trials included in the analysis was relatively small, the model fit of the nCREANN algorithm demonstrated excellent performance and strong generalizability from training to testing data. The connectivity patterns and source-localized regions identified here can serve as hypotheses for future confirmatory studies. These may use modified experimental designs and complementary validation techniques, such as structural connectivity constraints, perturbational methods (e.g., TMS-EEG).

In summary, we provide deepened insights into cortical theta, alpha, and beta band activity in managing perception-action (S-R) associations. Our findings emphasize the critical role of alpha band activity in sustaining these associations over time, with directed connectivity between regions like the insula and ventral visual areas supporting the influence of recent experiences on adaptive behavior. This connectivity reflects top-down control and the selective maintenance of task-relevant information, aligning with ideomotor principles of binding and retrieval. Unlike alpha, theta band activity appears essential for organizing sequential information but does not maintain continuity in S-R associations across time. These results offer advanced conceptual depth by illustrating how distinct oscillatory patterns in the brain manage dynamic S-R bindings, revealing a neural basis for how the immediate past shapes action control.

## Methods

### Participants

Overall, *N* = 49 participated in the present study, and *N* = 43 participants were included in the final data set. During an initial telephone screening, none of the participants reported any psychiatric or neurological illnesses. Via an online questionnaire on SoSci^57^, the participants filled out the Adult Self-Report for ages 18–59 (ASR^58^), a tool used to screen for potential psychiatric difficulties. Based on version B of the Mehrfach-Wortschatz-Intelligenztest (MWT-B^59^), all participants had at least an average IQ. Additionally, the Alcohol, Smoking and Substance Involvement Screening Test (ASSIST^60^) was carried out. After excluding five participants due to scores above the cut-off values in the ASR or the ASSIST, or who did not meet the inclusion criteria, and one participant due to technical difficulties, the final sample consisted of *N* = 43 (19 females, mean age 23.95 years ± 3.08, age range 20–30). The participants were part of a sample in a larger study that was collected between April 2019 and June 2021. All participants gave written informed consent, and either a financial reimbursement or course credit was given for their participation. The ethics committee of the Faculty of Medicine of the TU Dresden approved the study. All ethical regulations relevant to human research participants were followed.

### Task

The established stimulus-response (S-R) paradigm^34^, which is shown in Fig. 1, was administered to the participants using Presentation® software (version 14.9, Neurobehavioral Systems, Inc., Berkeley, CA, www.neurobs.com). Throughout the task, three vertically aligned boxes (each 2.4 × 0.9 cm) were presented in a rectangle. At the beginning of each trial, a cue stimulus in the form of an arrowhead pointing either left of right was shown in the middle box. Thereafter followed the consecutive presentation of stimulus 1 (S1) and stimulus 2 (S2), both of which were either displayed in the top or bottom box, in either green or red color, and as either a vertical or horizontal bar. Thereby, the stimuli could share up to three different features: position (top or bottom), color (red or green), and orientation (vertical or horizontal), resulting in four different overlap levels (no feature overlap, one feature overlap, two feature overlap, and three feature overlap). In the no feature overlap condition, S1 and S2 had no features in common; in the full feature overlap condition, on the other hand, S1 and S2 were identical. Participants were asked to remember the direction of the cue and indicate it by pressing the corresponding control key (left or right) with the respective finger upon the presentation of S1, irrespective of the features of S1. If an erroneous response (R1) was given, the trial was repeated. At presentation of S2, participants were instructed to indicate the orientation of S2 by pressing the left control key for a horizontal bar and the right control key for a vertical bar, irrespective of color and position. The participants were thus asked to give two responses (R1 at S1 and R2 at S2), with R1 based on (the direction of) the previously presented cue and R2 based on (the orientation of) S2. Each trial entailed either a response repetition (the responses given at S1 and S2 were identical) or a response alternation (the responses given at S1 and S2 differed). The participants were informed that there would be no systematic association between the features of S1 and S2, or between R1 and R2. Regarding the timing of the stimuli presentation, the cue was shown for 1500 ms, followed by the presentation of a blank screen for 1000 ms. Subsequently, S1 was displayed for 500 ms, and S2 was shown for a maximum of 2000 ms or until a response was given. S1 and S2 were separated by a blank screen shown for a duration of 2000 ms. In total, the task was comprised of 384 trials, which were divided by inter-trial intervals, jittered between 1500 ms and 2000 ms and during which a fixation cross was shown in the middle box. The task consisted of 6 blocks, between which participants had the possibility to take a break.

The binding effects at the center of the current investigation are statistically reflected by an interaction of the factors “response” (repetition vs alternation) and “overlap” (overlap of 0 features, 1 feature, 2 features, or all 3 features between S1 and S2^2,11,34^). Because past research has established that these effects are most reliably shown when the no feature overlap and full feature overlap levels are contrasted in dependency of response level (repetition or alternation^7,13,61^), the partial feature overlap levels are not taken into consideration in the present analysis. This simplified analysis strategy still follows the basic logit of response x feature relation commonly used in binding studies^3^.

### EEG recording and pre-processing

The EEG data were acquired from 60 equidistantly arranged Ag/AgCl electrodes using a BrainAmp amplifier and the Brain Vision Recorder 1.2 software (Brain Products, Germany). *θ* = 58, *ϕ* = 78 and *θ* = 90, *ϕ* = 90 were predefined coordinates of the locations of the ground electrode and the reference electrode, respectively. The data was recorded at a sampling rate of 500 Hz, with impedances under a maximum of 5 kΩ. The preprocessing was carried out using Automagic^62^ as well as EEGLAB^63^ in MATLAB (The MathWorks, Inc., version R2021b). The first step consisted in the resampling of the data to 256 Hz in order to save working storage and memory space. Subsequently, channels without neurophysiological activity were discarded, and the data were re-referenced to an average reference. The PREP preprocessing pipeline^64^ and the EEGLAB clean_rawdata pipeline were implemented, with the former removing line noise at 50 Hz by using a multitaper algorithm and discarding noise elicited from bad channels. Subsequently, an average reference was applied. In order to detrend the EEG data, noisy and outlier channels were identified and removed by the clean_rawdata pipeline using a FIR high-pass filter of 0.5 Hz (order 1286, stop-band attenuation 80 dB, transition band 0.25–0.75 Hz). Epochs with particularly strong power (i.e., >15 SDs in relation to calibration data) were reconstructed using Artifact Subspace Reconstruction (burst criterion: 15^65^). Time windows that could not be reconstructed were discarded, followed by the application of a lowpass filter of 40 Hz (sinc FIR filter; order: 86^66^). Subsequently, electro-oculography artifacts were removed by applying a subtraction method^67^, and further remaining artifacts were identified and discarded using an independent component analysis (ICA) based on the Multiple Artifact Rejection Algorithm (MARA^68,69^). Components that showed artifacts related to cardiac activity were removed using ICLabel^70^. Finally, using a spherical method, previously discarded channels were interpolated. On average, 9.5 ± 2.9 channels were interpolated after preprocessing. To examine the time window of interest from 0 to 1000 ms post-stimulus (for both S1 and S2) that has also been used in previous studies on this task^6,13^, and avoid potential edge effects in the Supplementary Analysis 1, which uses the same segments, the data were segmented into short segments lasting from −1000 to 2000 ms around S1 and S2, respectively. In order to create a common spatial filter based on all S2 conditions and the S1 segments for the beamforming procedure (see “Directed connectivity analysis”), for the calculation of this common spatial filter the data were segmented into long segments of -4000 to 2000 ms around the presentation of S2, encompassing both S1 and S2 time windows. Only trials where R1 and R2 were correct were included in the analysis.

### Beamforming analyses

Dynamic imaging of coherent sources (DICS) beamforming as provided by the FieldTrip toolbox^71^ was carried out to determine the source activity of the relevant frequency bands within each condition^20^. To this end, the common spatial filter was created based on the long segments encompassing both S1 and S2 and was then applied to the time window of 0 to 1000 ms after S1 and S2 separately. Common spatial filters were estimated based on the cross-spectral density of a Fast Fourier Transformation (FFT) of the averaged theta (4–7 Hz), alpha (8–12 Hz), and beta (15–30 Hz) activity. These were then applied to each condition across all to-be-beamformed segments together. A grid (equal spacing of 5 mm) was established with the forward model template toolbox from FieldTrip, the basis of which constituted the standard Montreal Neurological Institute (MNI) space. The head model was established based on the geometrical and conductive properties of the head, based on a Boundary Element Method (BEM) volume conduction model of the head^72^. Spatial filters were calculated using a regularization parameter of *λ* = 5%. To consider increased noise concomitant to more distance from the sensors, the Neural Activity Index (NAI) was determined by dividing the source activity estimates by the respective estimates of local noise per voxel^73^. Subsequently, the “Density-Based Spatial Clustering of Applications with Noise Algorithm” (DBSCAN^35^) was employed as implemented in MATLAB to determine the largest activity clusters in the three frequency bands of interest across all conditions together. The top 1% of the power distribution for the regions classified in the Automatic Anatomical Labeling (AAL) atlas^74^ was established as the common threshold for the largest activity in a frequency band across all frequency bands. It was ensured that this threshold resulted in at least one cluster of voxels in each condition. Thereby, the DBSCAN algorithm identified adjacent voxels, with a minimum of five voxels per cluster and an epsilon of 1.5*grid edge length.

### Directed connectivity analysis

To investigate directed connectivity patterns among clusters in different frequency bands as established through the DBSCAN algorithm, we applied the nCREANN method (non-linear Causal Relationship Estimation by Artificial Neural Network^24^). Using a non-linear Multivariate Autoregressive (nMVAR) framework, nCREANN employs an artificial neural network (ANN) to evaluate directed connectivity among various regions. The nMVAR model generates current samples of brain regions from the interactions of their previous samples. The nMVAR model is crucial for capturing temporal causality, where the cause precedes the effect^75^. Unlike traditional linear approaches, nCREANN accounts for linear and non-linear information flow dynamics between cortical areas. Since non-linear behaviors in the nervous system have been documented from single neurons to large-scale systems^76^, relying solely on linear methods may lead to an oversimplification of brain dynamics. Non-linear interactions play a significant role in the organization of information flow^77,78^. Multiple studies have highlighted the necessity of integrating both linear and non-linear principles to deepen our understanding of large-scale neuro-dynamics^79–82^.

Before applying the neural network algorithm, the time series of activity was isolated from the regions of interest as defined by the DBSCAN clustering. To this end, a linearly constrained minimum-variance (LCMV^73^) beamforming approach was applied to the segmented EEG data. Similarly to the DICS beamforming, the common spatial filter was created based on the segments encompassing the presentation of S1 and was subsequently applied to the shorter segments around the S2 stimuli. The activity time course was then band-pass filtered with respect to the frequency bands of interest.

In this model, the current samples of the signals are represented as a (non)linear function of their previous values, allowing for an inference of temporal causality. For a given time series ${{{\bf{x}}}}\left(n\right)\in {{\mathbb{R}}}^{M}$ with length *L*, a non-linear MVAR model of order *p* is expressed as:

1 $${{{\bf{x}}}}\left(n\right)={{{\boldsymbol{f}}}}({{{{\bf{x}}}}}_{p})+{{{\boldsymbol{\sigma }}}}(n)$$

Here, ${{{{\bf{x}}}}}_{p}={\left[{x}_{1}\left(n-1\right),{x}_{2}\left(n-1\right),\,\cdots ,{x}_{M}\left(n-p\right)\,\right]}^{{{{\rm{T}}}}}$ is a vector of *p* past samples, and ${{{\boldsymbol{\sigma }}}}\left(n\right)=\,{\left[{\sigma }_{1},\,{\sigma }_{2},\,\ldots ,\,{\sigma }_{M}\,\right]}^{T}$ represents the noise residuals. The function ${{{\boldsymbol{f}}}}$ delineates how past samples lead to future values. In nCREANN, ${{{\boldsymbol{f}}}}$ is divided into linear and non-linear components:

2 $${{{\boldsymbol{f}}}}={{{{\boldsymbol{f}}}}}^{{Lin}}+{{{{\boldsymbol{f}}}}}^{{NonLin}}$$

The linear component ${{{{\boldsymbol{f}}}}}^{{Lin}}$ provides estimates of Linear Connectivity $({{lC}}_{i\to j})$ as the linear impact of *i*th region on the *j*th region, while the non-linear component ${{{{\boldsymbol{f}}}}}^{{NonLin}}$ quantifies the non-linear connectivity $({{NC}}_{i\to j})$ from ${x}_{i}$ to ${x}_{j}$.

In this study, nCREANN was applied to the time courses of the beamforming-derived sources across five conditions: post-S1 time window, no overlap alternation, full overlap alternation, no overlap repetition, and full overlap repetition. In the beta and theta frequency bands, some conditions only revealed one activity cluster (beta band: all conditions, theta band: no overlap alternation condition, full overlap repetition condition). Therefore, these conditions were not included in the connectivity analysis, since it requires at least two clusters. Data from 0 to 1000 ms after stimulus onset were selected for connectivity analysis, and all trials in each condition were concatenated to form a sufficiently long dataset for network training. The optimal model order was determined to be *p* = 5 based on Akaike and Schwartz criteria^83,84^ and applied uniformly across conditions. A Multilayer Perceptron neural network with one hidden layer containing 10 neurons was trained, using ${{{{\bf{x}}}}}_{p}$ as input to predict ${{{\bf{x}}}}\left(n\right)$. The training employed gradient descent error back-propagation (EBP) with momentum (*α*) and an adaptive learning rate (*η*). To enhance generalization, an early stopping approach was utilized. A 10-fold permuted cross-validation method was implemented, dividing the dataset into 80% for training, 10% for validation, and 10% for testing. The network parameters were initialized randomly within the range of [−0.5, 0.5] and updated incrementally with each input sample. The nMVAR model’s validity was assessed using MSE and *R*² values. *R*² values close to 1 and low MSE close to 0 indicate good fit and performance, with similar values across training and test sets showing strong generalization. To determine the significance of the connectivity values, we performed a randomization test using 100 datasets generated with the time-shifted surrogate technique^85^. This technique eliminates causal effects between signals while preserving the inherent dynamics of each time series. Both driver and response variables were resampled, which has been shown to outperform in dealing with spurious connections^86^. The network parameters were maintained identically to those applied to the original data when analyzing the surrogate data using nCREANN. Based on the surrogate data, some individual connectivity values were deemed non-significant. Consequently, there were variables including missing data, and the respective analyses were only carried out with those subjects with no missing data in the required variables.

### Statistics and reproducibility

All statistical tests were carried out with the final sample of *N* = 43 participants. Statistical calculations were performed using IBM SPSS Statistics for Windows (version 27.0).

The behavioral data was analyzed statistically by means of repeated-measures ANOVAs in a within-subject design with the hit rate (i.e., number of correct responses divided by total number of events, including misses and false responses) and the reaction time (i.e., the time passed between stimulus presentation and response, where the mean for each condition is calculated per participant) as the measures of interest. The factors included in the ANOVA were “response” (repetition vs alternation) and “overlap” (no overlap vs full overlap). When significant interaction effects were established, post-hoc analyses were conducted using two-tailed paired-samples t-tests. Similarly, also any other comparisons were conducted using two-tailed paired-samples *t*-tests. In the event of a lack of a normal distribution as determined by a Kolmogorov–Smirnov test, a non-parametric test was used (Wilcoxon signed-rank test).

For the connectivity values, Spearman correlations were conducted to examine the relationships between the connectivities in the post-S1 time window and post-S2 conditions for the alpha and the theta band, respectively. Given the large number of correlations, the False Discovery Rate (FDR) correction was applied to control for Type I errors. We employed the Benjamini–Hochberg procedure at a significance level 0.05^36^, establishing critical values for the respective analyses.

### Reporting summary

Further information on research design is available in the Nature Portfolio Reporting Summary linked to this article.

## Supplementary information

Supplemental Material

Description of Additional Supplementary Materials

Supplementary Data 1

Reporting Summary

## Data Availability

Supplementary data 1 and other data can be downloaded from here: 10.17605/OSF.IO/79UDF^87^.

## Code availability

Further custom code used to process the data can be found here: 10.17605/OSF.IO/79UDF^87^.
