# Supplementary material for: Uncovering the role of directed connectivity in alpha and theta band activity for sustaining perception-action links
Source: Commun Biol. 2025 Aug 2;8:1147. doi: 10.1038/s42003-025-08601-y (PMC12317987; doi:10.1038/s42003-025-08601-y)
Supplement: Supplementary file 1 — Supplemental Material [file 42003_2025_8601_MOESM1_ESM.pdf]

Supplementary Information

Uncovering the Role of Directed Connectivity in Alpha and Theta Band Activity for Sustaining Perception-Action Links

Elena Eggert, Astrid Prochnow, Nasibeh Talebi, Christian Frings, Alexander Münchau, Christian Beste

Supplementary Analysis 1: Calculation of Binding Effects on the Neurophysiological Level

1. Methods

1.1 Time-frequency decomposition and cluster-based permutation tests

A time-frequency decomposition was carried out within the short segments of the EEG data by using Morlet wavelets with a width of seven cycles and a Gaussian kernel of 3. The range of the selected time frequencies was 3 Hz to 30 Hz (in steps of 1 Hz), because the predetermined theta (4-7 Hz), alpha (8-12 Hz), and beta (15-30 Hz) frequency bands were at the focus of the research question. For the time window of interest of 0 to 1000 ms after the S2 stimuli, cluster-based permutation tests were calculated for the results of the time-frequency decomposition in each of the three relevant frequency bands to contrast the different conditions using FieldTrip[1] (e.g., no overlap vs full overlap in the repetition trials, no overlap vs full overlap in the alternation trials). Clusters were deemed as such when the paired-samples t-test yielded a significant *t*-value ($p < .050$) in the sample itself as well as in at least two neighboring samples (i.e., two consecutive time points or two adjacent EEG channels). To estimate the reference distribution of the permutation test, 1000 draws were carried out using the Monte-Carlo approach. A cluster was considered significant when the *p*-values were below a critical alpha level of $p = .050$. Cluster-based permutation tests were calculated once averaged across time and once without averaging across time to also take more specific effects into account.

1.2 Beamforming analyses

Dynamic imaging of coherent sources (DICS) beamforming was carried out to determine the source activity of the relevant frequency bands within each condition[2]. To this end, the common spatial filter was created based on the long segments encompassing both S1 and S2 and was then applied to the time window of 0 to 1000 ms after S1 and S2 separately. Common spatial filters were estimated based on the cross-spectral density of a Fast Fourier Transformation (FFT) of the averaged theta (4-7 Hz), alpha (8-12 Hz), and beta (15-30 Hz) activity. These were then applied to each condition across all to-be-beamformed segments together. A grid (equal spacing of 5 mm) was established with the forward model template toolbox from FieldTrip, the basis of which constituted the standard Montreal Neurological Institute (MNI) space. The head

model was established based on the geometrical and conductive properties of the head, based on a Boundary Element Method (BEM) volume conduction model of the head[3]. A decibel conversion was performed to normalize the power values. Subsequently, the "Density-Based Spatial Clustering of Applications with Noise Algorithm" (DBSCAN[4]) was employed as implemented in MATLAB to determine the largest activity clusters in the three frequency bands of interest across all conditions together. The top 1% of the power distribution for the regions classified in the Automatic Anatomical Labeling (AAL) atlas[5] was established as the common threshold for the largest activity in a frequency band across all frequency bands. It was ensured that this threshold resulted in at least one cluster of voxels in each condition. Thereby, the DBSCAN algorithm identified adjacent voxels, with a minimum of five voxels per cluster and an epsilon of 1.5*grid edge length.

2. Results

When the data was not averaged across time points, the cluster-based permutation tests revealed a significant difference between no overlap and full overlap in the repetition trials in form of a positive cluster from 0 to 532 ms (T_{sum} = 2000.5, p = .044) in the theta frequency band. Based on DICS beamforming, this contrast was reflected by activity modulations on the source level in the supplementary motor area (SMA; BA 6). In the alpha frequency band, the results indicated a significant difference between the alternation and repetition trials in the full overlap condition, showing a negative cluster in the time window of 465 to 1000 ms (T_{sum} = -4963.9, p = .006). On the source level, DICS beamforming revealed activity modulations in the superior frontal (BA 8) and inferior frontal (BA 11) regions as well as in the temporal pole (BA 38). When the data was averaged across all time points (0-1000 ms), the cluster-based permutation test revealed a positive cluster denoting a significant difference between no overlap and full overlap in the alternation trials (T_{sum} = 71.4, p = .002) in the beta frequency band. Based on DICS beamforming, beta activity modulations for this contrast were shown in the middle frontal (BA 44) as well as the middle and inferior frontal regions (BA 45, BA 46). Furthermore, there was a negative cluster showing a significant difference between the alternation and repetition trials in the full overlap condition (T_{sum} = -107.8, p = .004) in the beta frequency band. Reconstructing source-level activity for this contrast, activity modulations were shown by DICS beamforming in the middle temporal regions (BA 21, BA 22) and the superior temporal regions (BA 22). The results of the time-frequency analysis, cluster-based permutation tests and beamforming are shown in Supplementary Figure 1.

To summarize, significant differences were observed in brain activity patterns depending on task overlap and trial type. These effects (reflecting binding effects) revealed that in the theta band, there was a significant positive cluster for no overlap versus full overlap in repetition trials in the supplementary motor area (SMA). In the alpha band, a negative cluster emerged between alternation and repetition in full overlap trials, linked to frontal and temporal regions was evident. In the beta band, contrasting alternation and repetition trials in full overlap showed significant clusters with modulations in middle and superior temporal areas.

3. Discussion

The neurophysiological data contrasting overlapping and non-overlapping S-R associations in the post-S2 interval revealed theta band activity modulation in the SMA. This modulation likely reflects the reconfiguration of perception-action associations[6–8] since theta activity was higher when the S2 stimulus features only partially overlapped with those of the S1. The pattern was reversed for alpha and beta band activity in the insular cortex and ventral visual stream regions: Activity was higher in conditions that did not entail the reconfiguration of a previously established S-R association. Alpha band activity is involved in the inhibitory gating of information[9], so increased alpha band activity prevents that other information is considered and that a reconfiguration occurs accidentally. Beta band activity possibly represents the status quo to maintain the current sensorimotor set[10] or content-specific changes from active to latent to re-activated states[11]. When there is no necessity to reconfigure perception-action associations, information gating is not required and the sensorimotor set does not have to be changed. Therefore, it is reasonable to assume that alpha and beta band activity was higher when the same information had to be maintained. These results align with previous studies' findings using the same paradigm[7,8] and other tasks[12,13].

Supplementary Figure 1: Neurophysiological Results of Contrasting S2 Conditions

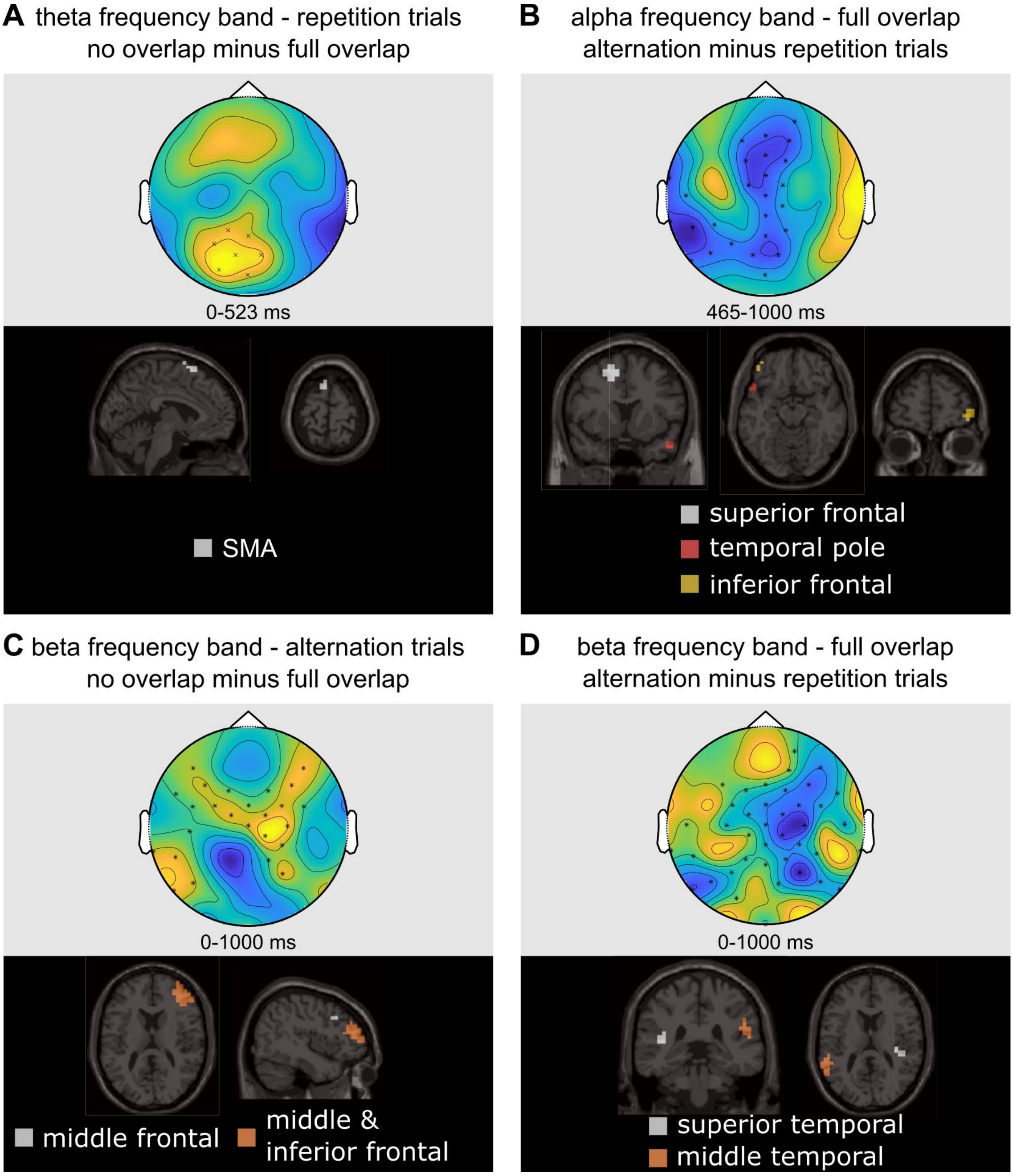

Contrasts of (A) no overlap minus full overlap conditions in repetition trials in theta frequency band and (B) alternation minus repetition trials in the full overlap condition in alpha frequency band. The topographic cluster plot (upper panel) shows results of a cluster-based permutation test at sensor level, conducted for illustration purposes and focusing on the significant time window and electrodes of the original test. The lower panel highlights brain regions with the top 1% highest differences. Contrasts in beta frequency band of (C) no overlap minus full overlap conditions in alternation trials and (D) alternation minus repetition trials in the full overlap condition. The upper panel shows the topographic cluster plot of the results of the cluster-based permutation test on sensor level, the lower panel highlights brain regions with the top 1% highest differences.

Supplementary Analysis 2: Comparing Directed Connectivity Strength Across Conditions in the Alpha Frequency Band

1. Methods

We examined in how far the directed connectivity strength varied across conditions that also constitute the binding effect. To this end, we focused on the post-S2 time interval. As described in section 4.5 of the main manuscript, some variables contained missing data after the surrogate data analysis. Only the subjects with complete data for respective variables were included in the repeated-measures ANOVA with the factors "connectivity direction", "response" and "overlap".

2. Results

With respect to the linear directed connectivity, n = 42 subjects were included. The repeated-measures ANOVA for all post-S2 conditions yielded a significant main effect of "response" ($F(1,41) = 8.74$, $p = .005$, $\eta_p^2 = .176$), with higher connectivity in repetition trials (.14 ± .05) than in alternation trials (.13 ± .04). Moreover, the interaction of "response" and "overlap" was established ($F(1,41) = 5.69$, $p = .022$, $\eta_p^2 = .122$). However, post-hoc Wilcoxon tests did not reveal significant differences between the overlap levels neither in alternation trials (no overlap: .12 ± .05; full overlap: .13 ± .05; $Z = -1.94$, $p = .052$) nor in repetition trials (no overlap: .15 ± .06; full overlap: .14 ± .05; $Z = -1.51$, $p = .132$). Other main effects or interactions did not reach significance ($F \leq 2.53$, $p \geq .119$).

With regard to the nonlinear directed connectivity, n = 42 subjects were included. The repeated-measures ANOVA for all post-S2 conditions yielded a significant main effect of "response" ($F(1,41) = 5.02$, $p = .031$, $\eta_p^2 = .109$), with higher connectivity in repetition trials (.21 ± .11) than in alternation trials (.17 ± .07). Moreover, the interaction of "overlap" and "connectivity direction" was established ($F(1,41) = 7.64$, $p = .009$, $\eta_p^2 = .157$). Post-hoc Wilcoxon tests revealed a significant difference between the overlap levels for the connectivity from N1 to N2 (no overlap: .18 ± .10; full overlap: .21 ± .10; $Z = -2.03$, $p = .042$), but not for the connectivity from N2 to N1 (no overlap: .20 ± .10; full overlap: .18 ± .09; Z = -:78, $p = .435$). Other main effects or interactions did not reach significance ($F \leq 4.00$, $p \geq .052$).

Supplementary References

1. Oostenveld, R., Fries, P., Maris, E. & Schoffelen, J.-M. FieldTrip: Open source software for advanced analysis of MEG, EEG, and invasive electrophysiological data. *Comput Intell Neurosci* **2011**, 156869 (2011).
2. Gross, J. *et al.* Dynamic imaging of coherent sources: Studying neural interactions in the human brain. *Proc. Natl. Acad. Sci. U.S.A.* **98**, 694–699 (2001).
3. Oostenveld, R., Stegeman, D. F., Praamstra, P. & van Oosterom, A. Brain symmetry and topographic analysis of lateralized event-related potentials. *Clin Neurophysiol* **114**, 1194–1202 (2003).
4. Ester, M., Kriegel, H.-P., Sander, J. & Xu, X. A density-based algorithm for discovering clusters in large spatial databases with noise. in vol. 96 226–231 (1996).
5. Tzourio-Mazoyer, N. *et al.* Automated Anatomical Labeling of Activations in SPM Using a Macroscopic Anatomical Parcellation of the MNI MRI Single-Subject Brain. *NeuroImage* **15**, 273–289 (2002).
6. Elsner, B. *et al.* Linking actions and their perceivable consequences in the human brain. *Neuroimage* **17**, 364–372 (2002).
7. Beste, C., Münchau, A. & Frings, C. Towards a systematization of brain oscillatory activity in actions. *Communications Biology* **6**, (2023).
8. Rawish, T. *et al.* Neurophysiological processes reflecting the effects of the immediate past during the dynamic management of actions. *NeuroImage* **288**, 120526 (2024).
9. Klimesch, W. Alpha-band oscillations, attention, and controlled access to stored information. *Trends in Cognitive Sciences* **16**, 606–617 (2012).
10. Fries, P. Rhythms for Cognition: Communication through Coherence. *Neuron* **88**, 220–235 (2015).
11. Spitzer, B. & Haegens, S. Beyond the Status Quo: A Role for Beta Oscillations in Endogenous Content (Re)Activation. *eneuro* **4**, ENEURO.0170-17.2017 (2017).

12. Prochnow, A., Wendiggensen, P., Eggert, E., Münchau, A. & Beste, C. Pre-trial fronto-occipital electrophysiological connectivity affects perception–action integration in response inhibition. *Cortex* **152**, 122–135 (2022).

13. Talebi, N. *et al.* Neural mechanisms of adaptive behavior: Dissociating local cortical modulations and interregional communication patterns. *iScience* **27**, 110995 (2024).
