# Supplementary material for: Uncovering the role of directed connectivity in alpha and theta band activity for sustaining perception-action links
Source: Commun Biol. 2025 Aug 2;8:1147. doi: 10.1038/s42003-025-08601-y (PMC12317987; doi:10.1038/s42003-025-08601-y)
Supplement: Supplementary file 2 — Description of Additional Supplementary Materials [file 42003_2025_8601_MOESM2_ESM.pdf]

Description of Additional Supplementary Files

File name: Supplementary Data 1

Description: numerical source data for Figure 5
